# Laser‐preparation of geometrically optimised samples for X‐ray nano‐CT

**DOI:** 10.1111/jmi.12577

**Published:** 2017-05-15

**Authors:** J.J. BAILEY, T.M.M. HEENAN, D.P. FINEGAN, X. LU, S.R. DAEMI, F. IACOVIELLO, N.R. BACKEBERG, O.O. TAIWO, D.J.L. BRETT, A. ATKINSON, P.R. SHEARING

**Affiliations:** ^1^ Department of Chemical Engineering University College London London U.K.; ^2^ Department of Earth Sciences University College London London U.K.; ^3^ Department of Materials, Royal School of Mines Imperial College London London U.K.

**Keywords:** laser micro‐machining, lithium‐ion batteries, sample preparation, shale, solid oxide fuel cells, X‐ray tomography

## Abstract

A robust and versatile sample preparation technique for the fabrication of cylindrical pillars for imaging by X‐ray nano‐computed tomography (nano‐CT) is presented. The procedure employs simple, cost‐effective laser micro‐machining coupled with focused‐ion beam (FIB) milling, when required, to yield mechanically robust samples at the micrometre length‐scale to match the field‐of‐view (FOV) for nano‐CT imaging. A variety of energy and geological materials are exhibited as case studies, demonstrating the procedure can be applied to a variety of materials to provide geometrically optimised samples whose size and shape are tailored to the attenuation coefficients of the constituent phases. The procedure can be implemented for the bespoke preparation of pillars for both lab‐ and synchrotron‐based X‐ray nano‐CT investigations of a wide range of samples.

## Lay description

A novel way of making samples so that they can be successfully imaged with X‐rays has been developed. This process involves using highly focused lasers to mill away excess material, to leave cylindrical samples ready to be placed in the X‐ray beam. The X‐ray procedure investigated is known as X‐ray computed tomography and is the materials science equivalent of medical CT scanners found in most hospitals. The technique involves rotating the small pillar in the path between a laboratory X‐ray source and a detector, producing a number of images, each similar to a classical bone scan. Using a sophisticated mathematical procedure, these images are reconstructed into a three‐dimensional volume, giving information about the complex microstructure at the nanoscale. This has been applied to materials used for energy generation and a geological sample to illustrate the versatility and robustness of the preparation route.

## Introduction

Since the first practical use of X‐ray computed tomography by Hounsfield (1973), the technique has been applied with increasing frequency, particularly in the areas of clinical radiology and diagnostic medicine (Kalender, 2006). More recently, the implementation of full‐field modes coupled with high‐resolution X‐ray optics has maximised the accessible spatial resolution for materials science applications, enabling the imaging of submicrometre features (Banhart, 2008).

**Figure 1:**
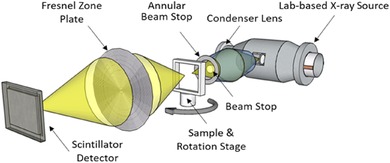
Schematic of the imaging set‐up used in typical lab‐based quasi‐monochromatic X‐ray nano‐computed tomography instrumentation.

A common system required for achieving high‐resolution tomography utilises wavelength‐selective Fresnel zone‐plates (FZP), which provide X‐ray focussing by means of a diffraction‐based lens composed of concentric rings with gradually smaller spacings towards the outer edge. These X‐ray optics are combined with either high‐brightness, highly collimated X‐ray radiation produced at third‐ or fourth‐generation synchrotrons, or with a cone‐beam lab‐source along with a capillary condenser lens, giving a focused pseudo‐monochromatic beam (Fig. 1).

When using a synchrotron source, which has inherently higher X‐ray flux, thicker sample volumes can be successfully imaged within reasonable exposure times (Laurencin *et al*., 2012), due to a higher signal‐to‐noise ratio than that afforded by conventional X‐ray tube sources in the same timeframe (Salvo *et al*., 2003). However, the fixed field‐of‐view (FOV) associated with nano‐CT systems in both lab‐based and synchrotron techniques means that there is a general need for samples to have submillimetre dimensions and to be appropriately X‐ray transmissive. By fabricating samples of this size, practical scanning times and sufficiently high signal‐to‐noise ratios required for reliable post‐acquisition analysis become achievable even on lab‐based instruments, and concomitant imaging artefacts are reduced. The problems of sample size and shape have been particularly acute in lab‐based systems to date (Maire & Withers, 2014), necessitating a cost‐effective and reproducible solution for the continued exploitation of X‐ray nano‐CT imaging.

Historically, crude and irreproducible forms of sample preparation have been implemented, which involve the fixation of mechanically sectioned sample pieces to a suitable substrate (pin, stub, etc.). Not only does this procedure lend itself to low throughput, as it is nontrivial to fabricate an appropriately sized sample, but it also leads to an underuse of the X‐ray FOV. In this paper, a new method is presented, which uses laser micro‐machining to provide suitably small and geometrically optimised samples for the X‐ray nano‐CT investigation of a range of materials and is illustrated with case studies from energy and geological research domains.

X‐ray nano‐CT has been widely applied to many materials, such as the three‐dimensional microstructure of solid oxide fuel cell (SOFC) electrodes (Shearing *et al*., 2013); lithium‐ion battery (LIB) electrodes (Harris & Lu, 2013) and geological samples (Cnudde & Boone, 2013), among others. However, in most cases, sample preparation has proved nontrivial, in that appropriately sized and suitably shaped samples for the X‐ray FOV are not reliably obtained by mechanical means. The first examples of applying X‐ray nano‐CT to the investigation of SOFC electrode microstructures were on lab‐based instruments, utilising phase and absorption contrast modes, and were conducted by Tkachuk *et al*. (2007) and Izzo *et al*. (2008). A rotating copper anode X‐ray source operating at 8 keV was coupled with the optical set‐up shown in Figure 1 to give a voxel dimension of 42.7 nm (with the addition of a Zernike phase ring downstream from the sample for phase imaging). From an anode‐supported tubular SOFC, a small piece was mechanically removed, comprising part of the solid electrolyte layer and part of the porous anode layer. This crude preparation lacks specificity in sampling volume, gives an irreproducible size and irregular shape, as well as potentially suffering from nonlocalised damage. As a result, an exposure time of 300 s was needed, which can be considered both impractical for routine 3D characterisation and highly susceptible to stage and temperature shifts throughout scanning.

Later work, conducted by Shearing *et al*. (2009), utilised a focused‐ion beam (FIB) lift‐out sample preparation technique to fabricate a sample geometry to remain inside the FOV throughout the scan. Despite having high spatial resolution (32 nm isotropic voxels), only a binary segmentation was possible, separating the two solid phases, nickel and yttria‐stabilised zirconia (YSZ), from the pore phase. The inability to separate Ni from YSZ is thought to be due to a low signal‐to‐noise ratio due to relatively large sample thickness.

To nondestructively obtain information about the triple‐phase boundary (TPB – the one‐dimensional interface at which all three constituent phases meet) in these electrodes, most studies have been carried out at a synchrotron, with the added benefits of higher flux and tuneable energy (e.g. Grew *et al*., 2010a,b; Shearing *et al*., 2010; Shearing *et al*., 2011). The higher throughput at synchrotron systems also demonstrates the potential for 4D (3D plus time) *in situ* measurements. However, fragile materials cannot be prepared by this method due to the force exerted during mechanical sectioning and the FIB lift‐out procedure. For example, with the latter technique, Pt welding is commonly used to attach the small sectioned sample to a micromanipulator for transition between instruments, and this is, unfortunately, liable to fail both on preparation and during heating (Shearing *et al*., 2012). Moreover, many of the aforementioned studies performed image analysis on volumes smaller than 700 μm^3^ due to underuse of the FOV; this small volume may prove unrepresentative for a variety of microstructural parameters of interest (Joos *et al*., 2012).

More recently, investigations were carried out by Kennouche *et al*. which inspected the influence of annealing time on the agglomeration behaviour of nickel in SOFC anodes (Kennouche *et al*., 2016a,b), and successfully registered image data from tomographs taken after *ex‐situ* heating. The sample preparation constituted four laborious steps, two of which were performed using a FIB instrument, which is both costly and time‐consuming.

X‐ray nano‐CT has also been used to study various other materials in the sectors of energy and geology where sample preparation techniques are also challenging and nonideal. For example, the active materials in LIB electrodes are deposited onto metallic current collectors that are typically removed prior to scanning to maximise signal‐to‐noise – this has been achieved by acid dissolution or mechanical methods, both of which may affect the electrode microstructure. This requirement is particularly acute when phase contrast enhancement is used due to inherently lower photon counts and because limits to fabricating suitably narrow Zernike phase contrast rings constrains these imaging procedures to the use of low energy beams (Kashkooli *et al*., 2016; Taiwo *et al*., 2016b).

Preparation techniques of natural rock materials are also challenging (Cnudde & Boone, 2013; Bin *et al*., 2013) where maintaining the materials’ delicate microstructure is essential for accurate measurement of material properties. For example, Gelb *et al*. (2011) imaged shale samples at both the micro‐ and nanoscale – wherein sample preparation is described as ‘physical extraction’ – and identified the minimum sample volume (17 000 μm^3^) required for extracting material properties that are representative of the bulk. However, further multiscale work by Bin *et al*. (2013), which characterised pore throats in tight sandstone samples, gives no information about sample preparation for ultra‐high resolution X‐ray CT.

Micro‐ and nanoscale studies have also been carried out with synchrotron X‐ray sources, such as the quantitative analysis performed by Sayab *et al*. (2016) on orogenic gold at voxel dimensions as small as 50 nm. Sample preparation for nano‐CT entailed a mixture of mechanical crushing and high‐voltage pulses in order to access large arsenopyrite crystals. However, such procedures are liable to damage delicate geological features in many rocks of interest, and often lead to geometries that underuse the FOV. The most recent work in this field has centred on multilength‐scale X‐ray nano‐CT and 3D‐electron microscopy (3D‐EM) analysis of carboniferous Bowland shale, which highlighted the difficulty of ultramicrotomy due to the high brittleness of shale (Ma *et al*., 2016).

In summary, there is a clear need for an improved sample preparation technique that can preserve the material microstructure and achieve optimum sample geometries for the FOV of nano‐CT scans. This paper illustrates the scope of a laser‐preparation technique, highlighting the importance of considering the individual attenuation power of constituent phases so as to fine‐tune sample thickness, and seeks to underline some specific advantages garnered from fabricating fine cylindrical pillars of different materials.

## Sample requirements

Good image quality, which renders subsequent segmentation more reliable, derives from an adequately high signal‐to‐noise ratio and is achieved by matching the sample to the FOV. Moreover, as the FOV is fixed, any part of the sample outside of this area is not imaged, leading to artefacts on reconstruction. Hence, control of sample size, particularly down to sizes smaller than the FOV, is essential. In some cases, this is relaxed to slightly larger (for lower attenuating materials) samples where oversampling projections can compensate for these artefacts. Other materials, such as highly attenuating metals and ceramics, may require preparation that gives a sample with dimensions significantly smaller than the FOV to obtain adequate transmitted flux.

This paper outlines a technique for fabricating optimal geometry samples by producing uniform cylinders and balancing the need for a large representative volume with a small enough thickness to provide sufficient photon counts on the scintillator‐detector. Assessing the optimal thickness can be approached by considering the attenuation power of individual phases within composite materials.

The degree to which a nondiverging monochromatic incident beam is attenuated is a form of the Beer‐Lambert Law, given below (Eq. (1)).

(1) $$I\;=\;I_{0}\exp\left[-\int \left(\left(\frac{\mu \left(L\right)}{\rho }\right)\rho \right)\mathrm{dL}\right],$$

where *I* is the transmitted X‐ray intensity, *I_0_* is the incident X‐ray intensity, μ/ *ρ* is the mass attenuation coefficient at a given energy, *ρ* is the mass density and *L* is the path length. Accounting for the constituent mass densities (*ρ_i_*), the attenuation lengths (*x_i_*) ascribable to each material can be calculated as per Eq. (2) (where μ is the attenuation coefficient), provided that the radiation is parallel and monochromatic.

(2) $$\begin{array}{ccc} \int \left(\left(\frac{\mu (L)}{\rho }\right)\rho \right)\mathrm{dL} & = & -\mathrm{In}\left|\frac{I}{I_{0}}\right|,\\ x & = & \frac{1}{\left((\mu /\rho )\rho \right)} \end{array}$$

The attenuation length is defined as the path length of material through which the radiation permeates before reducing to 1/*e* of its original intensity.

Thus, to enable readily accessible, high data quality collection, particularly with lab‐based sources, small samples are required. For example, it is worth noting that at an X‐ray energy of 5.4 keV, the theoretical path length/pillar diameter for a typical SOFC anode sample consisting of 33% porosity must be smaller than or equal to 30 μm to attain average transmission values above 5% and to provide sufficiently high signal‐to‐noise (for reliable segmentation) at practical exposure times (<100 s).

## Experimental

### Sample information

A planar SOFC anode, nominally comprised of equal volume fractions of nickel, YSZ and porosity, was supplied by Forschungszentrum Jülich (FZ, Germany), consisting of an approximately 550 μm thick anode support layer (ASL) and a much thinner anode functional layer (AFL), roughly 5 μm in thickness.

A Li‐ion battery cathode composed of a LiNi_1/3_Mn_1/3_Co_1/3_O_2_ (NMC) film material, which was supplied by Tagray (Montreal, Canada), was coated on an aluminium current collector (20 μm). The average thickness of the electrode layer was 55 μm. The active material particles were mixed prior to coating with Kynar HSV 900 binder and super C65 carbon in a % mass ratio of NMC:Kynar:C65  =  90:5:5.

A Li‐ion battery graphite anode with an average overall thickness of 95 μm, including a 20 μm copper current collector, was also studied. The electrode was composed of graphite, conductive carbon and two binders, with an approximate composition by volume of 63% graphite, 1% conductive carbon and 5% binder, where the remaining 31% of the volume was pore phase.

A clay‐rich shale sample, with an average mineral grain size of between 1 and 10 μm, was supplied by the Earth Sciences Department of University College London (UCL, London, UK). Shale rocks are laminated mudstones containing significant amounts of clay minerals ± kerogen, mixed together with silt‐sized carbonate and silicate minerals. The mineralogy of the sample is 70% clay (illite), 12% quartz, 6% muscovite, 5% feldspar, 4% dolomite and 3% pyrite. The laminated mineral arrangement of compositional layers and aligned platy clay minerals form the characteristic texture of shale rocks, which occurs at the micron scale.

### Laser procedure

An A Series/Compact Laser Micromachining System (Oxford Lasers, Oxford, UK) with an embedded Class 4, 532‐nm‐wavelength laser was used for all laser‐preparation of samples. The instrument has a maximum pulse energy of 1 mJ, normally functions between 0.1 and 2.4 W, and has a beam pulse duration between 10 and 500 ns. All work was carried out at 5000 Hz as this setup had been calibrated to give the most reproducible spot energy, size and shape. Laser power was adjusted according to the sample type and procedure followed.

**Figure 2:**
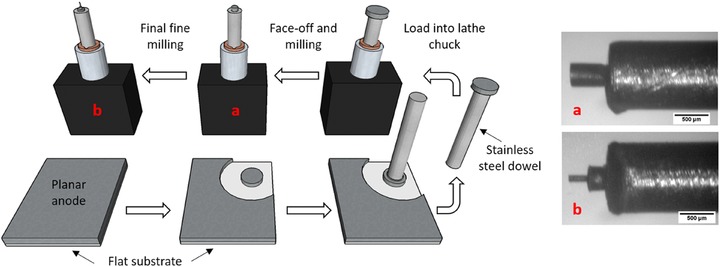
Workflow for sample preparation route and corresponding optical images for (A) base tier of structure and (B) fine pillar tier.

The protocols developed in order to fabricate pillars depended on the initial sample thickness. The process used to make high aspect ratio, mechanically stable pillars from thicker samples (SOFC anode and shale) is illustrated in Figure 2. A key strategy to ensure the structural integrity of these fine structures is to follow a tiered regime, thus reducing the stresses experienced at the fine end of the pillar. This involves milling a coarse pillar approximately 1 mm in diameter from a planar sample, often penetrating through the entire thickness, before securing the cored piece to a dowel with epoxy and allowing to cure for at least 10 min. The other end of the dowel is placed in a rotary chuck designed to mimic the behaviour of a mechanical lathe by rotation and movement under a fixed laser beam. The sample end is faced off and a tiered structure is machined by first reducing the pillar diameter to approximately the diameter of the dowel, before machining a shorter section to the fine pillar diameter, as dictated by the sample itself. This procedure provides a solid base for the fine pillar to be placed in the X‐ray beam.

Two SOFC samples were investigated, in each case by machining a coarse pillar of 1 mm diameter from planar substrates. At lower laser power, after fixation to a 600‐μm diameter dowel, each coarse pillar was refined down to a base pillar of 500 μm in height and diameter, before a fine pillar was fashioned out of the end farthest from the dowel. For the first sample, the fine pillar was 65 μm in diameter and 220 μm in height and for the second sample, a finer pillar of 30 μm in diameter and 285 μm in height were achieved, which was later transferred to the FIB for milling the top section to approximately 20–25 μm in diameter. Between each machining phase, X‐ray nano‐CT imaging was performed to assess the obtainable image quality.

**Figure 3:**
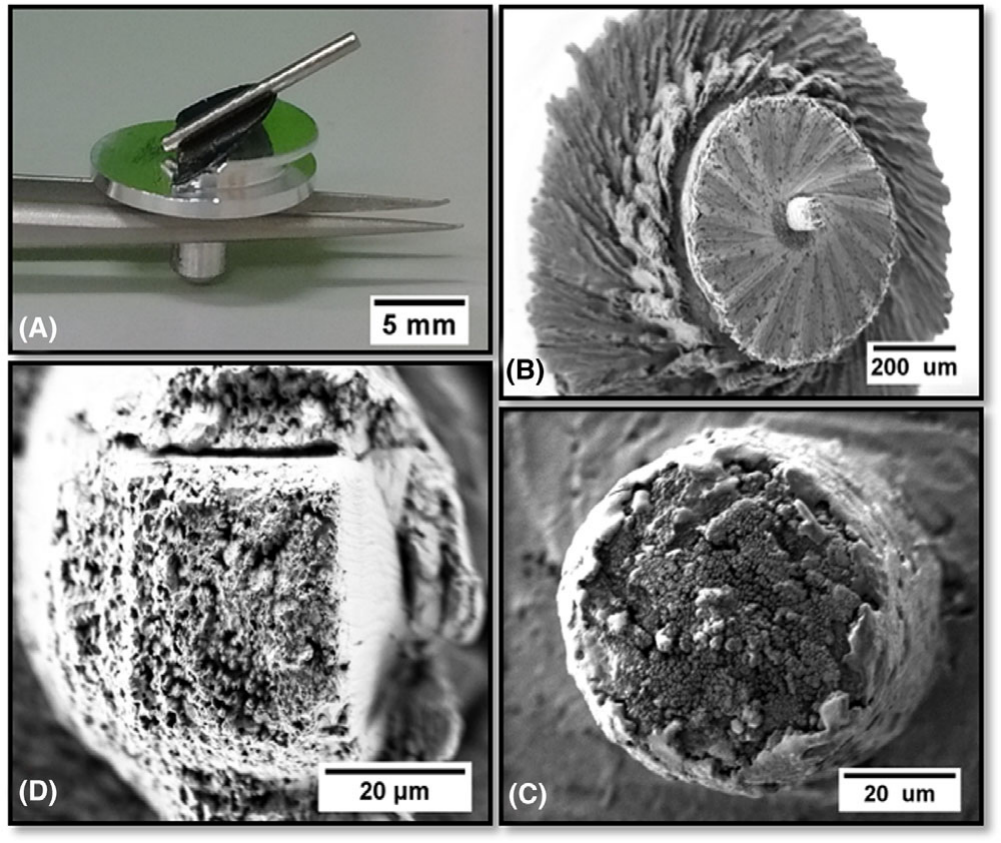
(A) Photograph of 36° Al stub with a dowel attached, SEM micrographs of (B) tiered structure at low magnification, (C) pillar aligned before milling and (D) pillar after FIB milling (clockwise from top‐left).

The secondary FIB milling was carried out using the Zeiss 1540XB CrossBeam (Carl Zeiss, Oberkochen, Germany) at the London Centre for Nanotechnology (LCN, London, UK). After laser micro‐machining to approximately 30 μm, the dowel was affixed to a special SEM stub (with an oblique surface angled at 36° to the horizontal) with sticky carbon tape before being gold‐coated and placed under vacuum in the instrument. This allowed easy alignment with the FIB column: all machining was undertaken at a milling current of 20 nA. The mounted stub, tiered structure and SEM micrographs of before and after FIB‐milling can be seen in Figure 3.

For the LIB samples, a tiered structure was not necessary, due to their thin film nature. In both cases, a 1 mm diameter coarse pillar was milled down to approximately 80 μm in increments of approximately 200 μm.

For the shale rock sample, first, a large pillar of approximately 2 mm in diameter was milled from a macroscopic substrate. Subsequent to X‐ray micro‐CT imaging, a pillar of approximately 65 μm in diameter was machined from this coarse pillar at lower laser power, atop two larger tiers (200–250 μm in diameter).

### X‐ray nano‐computed tomography (X‐ray nano‐CT)

X‐ray nano‐CT was performed on a Zeiss Xradia 810 Ultra, which had a fixed X‐ray energy of 5.4 keV. The FOV in all nano‐CT scans was 65 μm with voxel dimensions depending on the degree of binning. Binning corresponds to the summation and averaging of the photon counts of adjacent pixels, giving a new effective pixel size (bar binning 1). The value given for the binning corresponds to the dimensions of the box of adjacent pixels grouped together. For examples, binning 1 describes the maximum attainable resolution (approximately 63 nm) where every pixel contributes its value to the image and binning 2 corresponds to a quarter of the number of output pixels by the averaging of adjacent 2 × 2 boxes of pixels. This gives a resolution of 126 nm, with binning 4 corresponding to 252 nm. For the shale sample, X‐ray micro‐CT was performed on a Zeiss Xradia 520 Versa, with a polychromatic beam whose average energy was tuned to 70 keV.

Imaging parameters are shown in Table 1. A standard filtered back‐projection algorithm in the commercially available Zeiss XMReconstructor software was used to reconstruct three‐dimensional volumes from all projection data. The resulting stacks of two‐dimensional slices were processed in commercially available Avizo software (FEI, Hillsboro, Oregon, USA). A diffusive flux‐based approach was implemented in order to extract a tortuosity factor from relevant microstructural data, using an open source MATLAB software package known as TauFactor (Cooper *et al*., 2016). Representative volume element (RVE) was also computed where relevant, using porosity and tortuosity factor as the parameters of interest.

**Table 1:** Tabulated data displaying sample size and imaging conditions

		Imaging parameters			
Sample	Pillar width (μm)	Projections	Exposure time (s)	Binning	Details
SOFC anode	65	901/1601, 1601	60	2, 1	High projections warranted
	30	1401	50	1	Segmentation not possible
	60	2001	36	1	Laser only
	20–25	1601	64	1	Laser & FIB
LIB anode	80	2001	5	4	Absorption and phase
LIB cathode	80	1601	30	2	Laser only
Shale	2000^a^	1601	25	1	micro‐CT, pixel size 1.05 μm – data shown
	65	2001	36	1	Laser only

^a^Carried out on a polychromatic source with 70 keV average energy.

## Results and discussion

### Case study 1: SOFC

**Figure 4:**
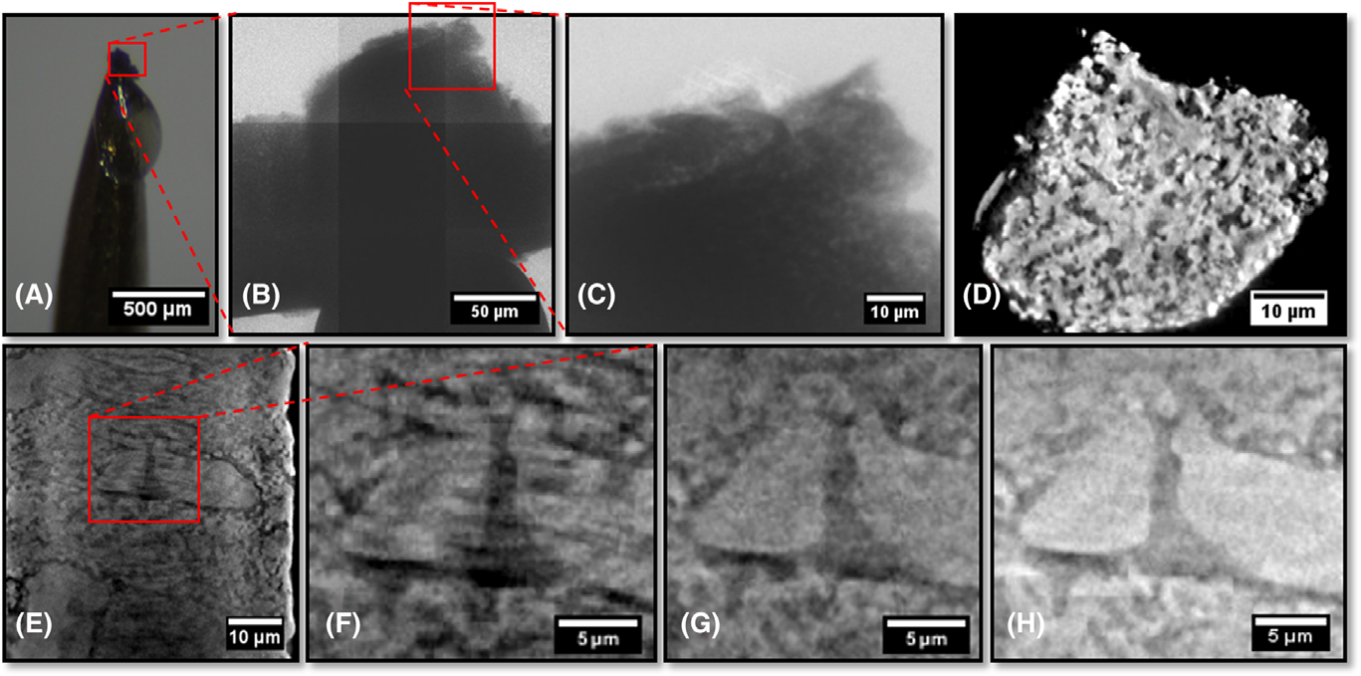
(A) Optical image of traditional mechanical preparation where the red square highlights the location of the sample material. (B) Corresponding mosaic of radiographs of the sample material. (C) Single radiograph with a FOV equal to that used for CT. (D) Single raw orthoslice from reconstructed tomogram. (E) Tomographic vertical slice from 60 μm pillar, magnified features at (F) 901 projections, binning 2, (G) 1601 projections, binning 1 and (H) 1601 projections, binning 2.

**Figure 5:**
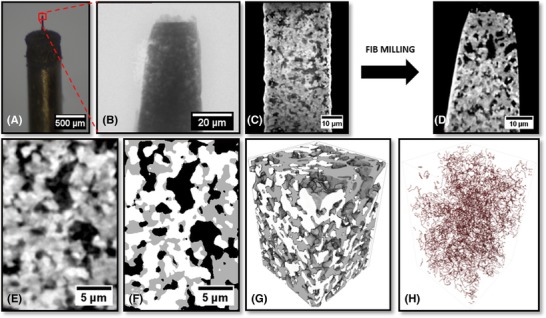
(A) Optical image of the new laser‐prepared pillar where the red square highlights the region of interest at the top of the pillar. (B) Corresponding radiograph of the top of the pillar. (C) Reconstructed vertical slice directly after laser micromachining. (D) Reconstructed vertical slice after further FIB milling. (E) Raw slice from usable volume after FIB milling. (F) Corresponding segmented image showing the three phases. (G) 3D reconstruction of full volume (white = YSZ, grey = nickel, transparent = pore volume), (H) 3D TPB map.

A visual comparison between the result of the typical mechanical process and an SOFC sample prepared by the laser technique is shown in Figures 4(A) and 5(A).

The pillar diameter was machined to fit the FOV (65 μm) and clear agglomerated features and the pore network were visible; however, image contrast between the solid Ni and YSZ was lacking (Fig. 4E), illustrating that further shrinking of the pillar was necessary to overcome signal‐to‐noise issues, both to improve the quality of the image and provide solid‐phase contrast. Moreover, it was shown that for good 3D data quality at least 1601 projections were necessary, and to achieve the maximum resolution of 63 nm (binning 1), the pillar would need to have a smaller diameter because the image was too noisy with a sample diameter of 60 μm (see Fig. 4G).

A second SOFC anode sample was laser micro‐machined initially to 30 μm diameter. In Figure 5(C), it is clear that the more attenuating YSZ (brighter) is distinguishable from the less attenuating nickel phase (darker), particularly in the centre of the image. Nevertheless, attempted segmentation led to difficulties in assigning these phases, hence FIB milling was employed to further reduce the sample thickness and provide a better signal‐to‐noise ratio (Fig. 5D).

Figure 5(E) shows an orthoslice from the vertical plane of the reconstructed 3D volume comprising 260 × 269 × 387 voxels, each with dimensions of 63.1 nm. The data were segmented by a mixture of grey‐scale and watershed thresholding without the requirement for image filtering. Manual adjustment at boundaries using an erosion and dilation approach was employed to improve the fidelity of segmentation. An example slice and a full 3D reconstruction can be seen in Figures 5(F) and (G).

Automated segmentation of the volume into three distinct phases (Ni, YSZ, pore) was possible giving rise to the microstructural parameters given below in Table 2, which closely matched the predicted ratio from the manufacturer (1:1:1). The absolute volume was calculated by a simple voxel counting algorithm and the volume‐specific surface area (VSSA) was calculated from the ratio of the total face area (computed by TauFactor; Cooper *et al*., 2016), barring that exterior to the subvolume, to the individual phase volumes.

**Table 2:** Absolute and relative volume and surface area data for each phase

Phase	Absolute volume (μm^3^)	Volume fraction	Volume specific surface area (μm^−1^)
Pore	2263	33.3%	1.38
Nickel	2327	34.2%	1.78
YSZ	2213	32.5%	1.45
Total	6802	100%	–

Interfacial properties (see Table 3) including the TPB density (*ρ*_TPB_) and volume‐specific interfacial areas (VSIA), were also extracted: the percolated TPB density was estimated to be 1.91 μm μm^−3^, which is in good agreement with recent literature values for state‐of‐the‐art Ni‐YSZ anodes (Kennouche *et al*., 2016a). A 3D percolated TPB network is shown in Figure 5(H).

**Table 3:** Interfacial information for the three phases in the SOFC anode

Interface	Absolute interfacial area (μm^2^)	Volume‐specific interfacial area (μm^−1^)	Volume‐specific length (μm^−2^)
Pore‐nickel	4200	0.62	–
Pore‐YSZ	2591	0.38	–
Nickel‐YSZ	4571	0.67	–
TPB	–	–	1.91

**Figure 6:**
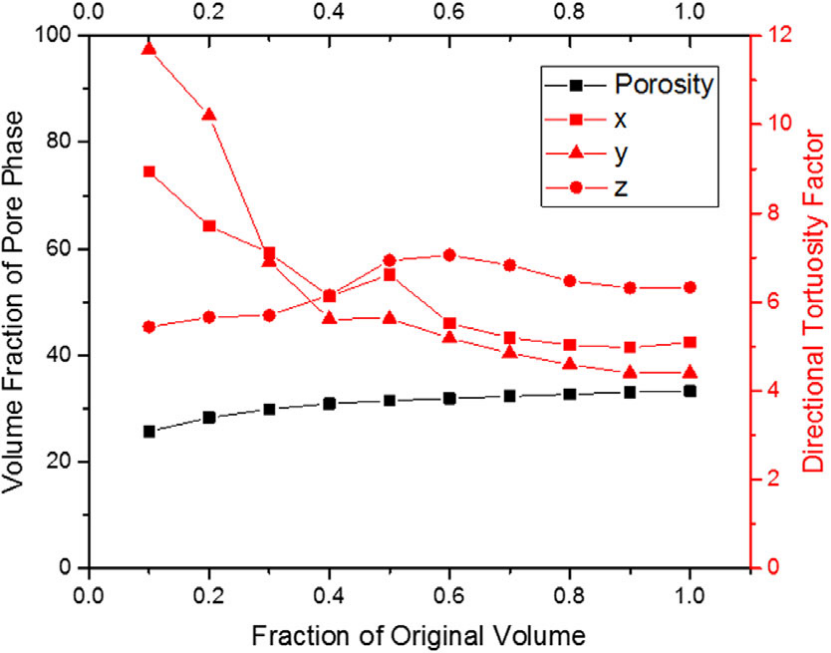
Plot displaying representative volume element analysis data for pore volume fraction and directional tortuosity factor (all three orthogonal directions).

Tortuosity factors for the pore phase in the three orthogonal directions were calculated using TauFactor, along with a representative volume element (RVE) analysis using both the pore volume fraction and tortuosity factor as the representative microstructural parameters (Fig. 6 **)**. The results are summarised in Table 4.

**Table 4:** Tortuosity factor and RVE analysis of the SOFC anode

Volume fraction		0.1	0.3	0.5	0.7	0.9	1.0
Porosity (%)		25.7	29.9	31.5	32.3	33.1	33.3
Tortuosity factor	Direction						
	*x*	8.94	7.11	6.62	5.20	4.98	5.09
	*y*	11.7	6.91	5.63	4.85	4.40	4.40
	*z*	5.44	5.70	6.94	6.83	6.32	6.33

The RVE analyses conducted here suggest a volume of approximately 4750 μm^3^ is necessary for representative sampling in terms of measuring the porosity of the sample and a volume approaching 5440 μm^3^ is necessary for representative sampling when considering tortuosity factor. Both these representative volume analyses illustrate the need for volumes of ca. 5000 μm^3^ for microstructures with submicrometre features, which is larger than most previously investigated SOFC electrode volumes (Joos *et al*., 2012) and requires maximisation of the X‐ray FOV.

### Case study 2: Li‐ion battery NMC cathode

To the authors’ knowledge, this is the first time cylinders of thin film battery electrodes have been prepared by a laser micro‐machining technique for investigation by X‐ray nano‐CT. For low‐atomic‐number materials, which attenuate the beam less than the metals and ceramics in the SOFC, it has been observed that the material outside of the FOV gives fewer artefacts when the truncated projections are reconstructed. As a result, oversampling projections is more capable of compensating for the otherwise obscuring features, allowing access to high‐quality image data for region‐of‐interest (ROI) scans. Using this approach, a sufficient signal‐to‐noise ratio can still be achieved, thereby maximising the usable volume, making it more representative, and avoiding the inclusion of the possibly damaged surface of the pillar. Consequently, this procedure constitutes a reliable and versatile approach to X‐ray nano‐CT sample preparation of lithium battery materials.

**Figure 7:**
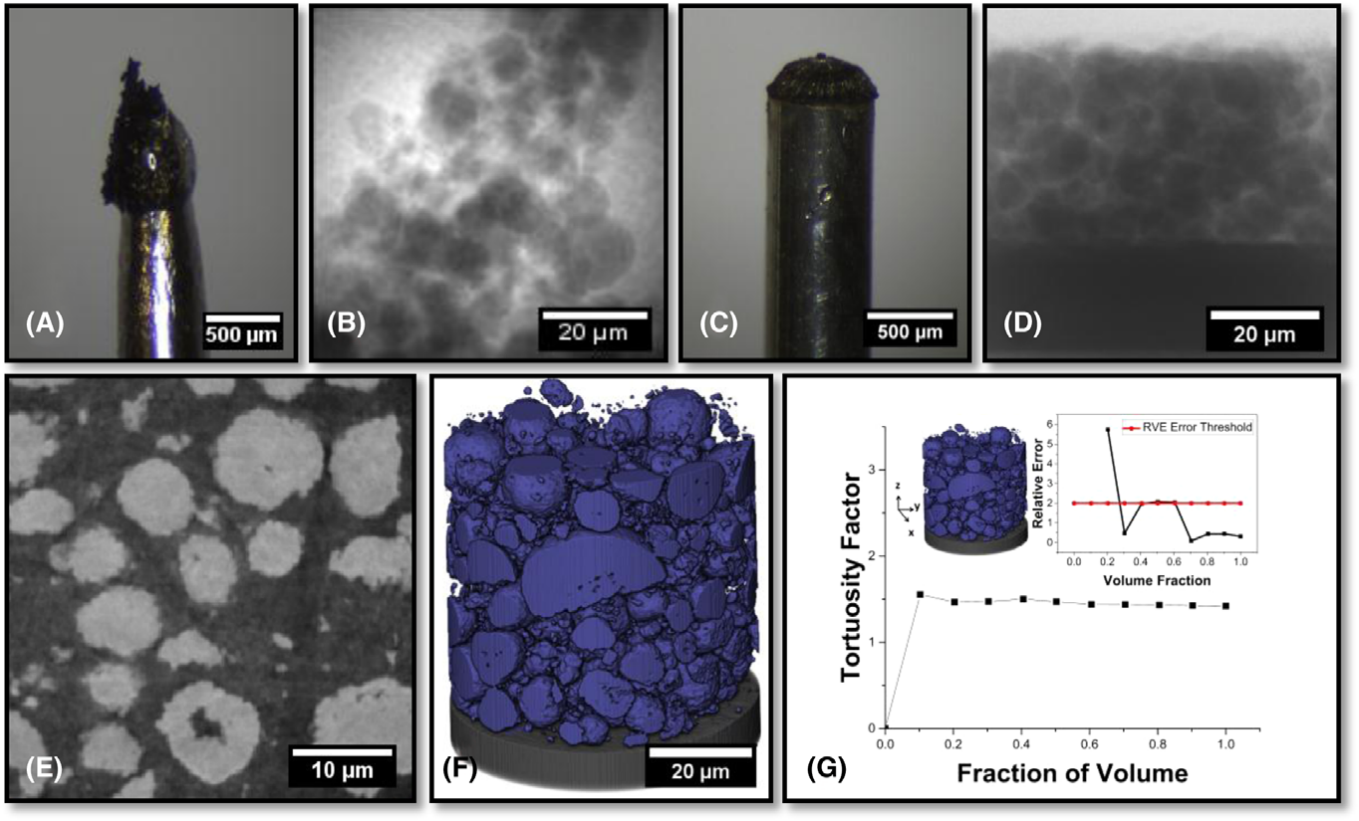
(A) Optical image of traditional mechanical preparation. (B) Corresponding radiograph. (C) Optical image of new laser‐prepared pillar. (D) Corresponding radiograph. (E) Raw orthoslice showing NMC electrode particles. (F) 3D surface rendering of electrode particles (blue) and current collector (black). (G) RVE Analysis of tortuosity calculated in the through‐plane direction, with error inset.

A significant benefit to this approach is that it renders the removal of the current collector unnecessary; traditionally the current collector is removed, possibly with concomitant mechanical damage. Furthermore, bulging and mechanical damage from sectioning with a sharp blade are avoided. With this laser‐preparation approach, the full electrode thickness may be captured (Fig. 7D), allowing not only for maximised volumes, but more importantly, for directionally significant information to be extracted. For example, tortuosity factor measurements, which cover the entire thickness (in the direction perpendicular to the electrode‐current collector interface), can be simulated and the results of this analysis are displayed in Figure 7(G).

Figure 7(E) shows an orthoslice from the reconstructed 3D volume comprising 500 × 512 × 511 voxels, each with dimensions of 126.2 nm. The data were segmented by simple grey‐scale thresholding without image filtering. An erosion and dilation approach was employed to remove the internal porosity within the particles. An example 3D reconstruction can be seen in Figure 7(F).

The microstructure was subdivided into particle, pore and current collector phases. The absolute volume was calculated by a simple voxel counting algorithm and the tortuosity factor was calculated for a subsection of the porous phase, 324 × 338 × 397, to minimise computation. The calculation was performed both in the direction perpendicular to the current collector (the direction of Li^+^ diffusion through the electrode during battery operation) and in both lateral directions, by solving Fick's first law of diffusion using TauFactor. The value for the tortuosity factor in the Li^+^ diffusion direction was 1.42 (see Fig. 7G), which is in keeping with recent literature (Ebner *et al*., 2014) for an electrode with a porosity of approximately 55%.

It is also observed that the lateral tortuosity factors (1.36 and 1.35) are very similar and slightly lower than for the *z*‐direction, also in keeping with the literature. The RVE analysis, with a marked threshold at 2% (Taiwo *et al*., 2016a), also indicates that this volume is sufficiently large to be representative of the electrode in terms of directional tortuosity factor; the results are presented in Table 5. The loading of the internal subsection was calculated to be 10.5 mg cm^−2^ which is in good agreement with the supplier value of 10 mg cm^−2^.

**Table 5:** Tortuosity factor and RVE analysis of LIB cathode

Volume fraction		0.1	0.3	0.5	0.7	0.9	1.0
Porosity (%)		55.6	56.9	57.2	57.2	57.4	57.4
Tortuosity factor	Direction						
	*x*	1.46	1.40	1.39	1.37	1.37	1.36
	*y*	1.49	1.39	1.40	1.37	1.35	1.35
	*z*	1.56	1.47	1.47	1.44	1.43	1.42

**Figure 8:**
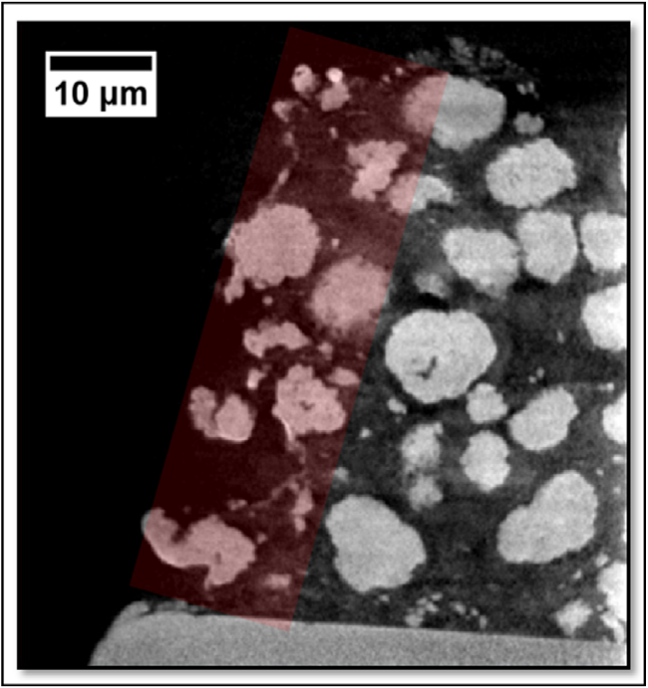
Vertical plane radiograph of the side of Li‐NMC electrode pillar, laser‐affected zone (LAZ) highlighted in red.

To investigate any detrimental effects of the laser on the samples produced for X‐ray imaging, the edge of a Li‐NMC pillar was placed in the FOV and the data analysed in order to inspect the extent of laser damage. As can be seen in Figure 8, there are deformed particles at the extremities of the pillar, but this only encompasses particles that are either directly on the surface, or one particle width into the sample from the surface. Therefore, this laser‐affected zone (LAZ) is an annulus, on the outer edge of the pillar, with a thickness of approximately 10 μm. Provided that the pillar width is at least 20 μm greater than the FOV, the imaged volume in a ROI scan will be predominantly free from LAZ artefacts, particularly as the usable volume is routinely cropped from the imaged volume. For a FOV scan, effective cropping can also be used; the LAZ is small enough not to significantly impact the analysis of the resulting data, which would remain relatively large and directionally intact. This reasoning is no doubt sample‐dependent, but in the case of Li‐NMC cathodes, the LAZ and unaffected regions are easily differentiated.

### Case study 3: Li‐ion battery graphite anode

A similar procedure was carried out for a commercial LIB graphite anode material, which was scanned using both lab‐based absorption and Zernike phase contrast X‐ray nano‐CT. In the case of the latter imaging mode, due to a reduced flux compared with the absorption imaging, sample size and signal‐to‐noise ratio is even more critical (Taiwo *et al*., 2016b).

**Figure 9:**
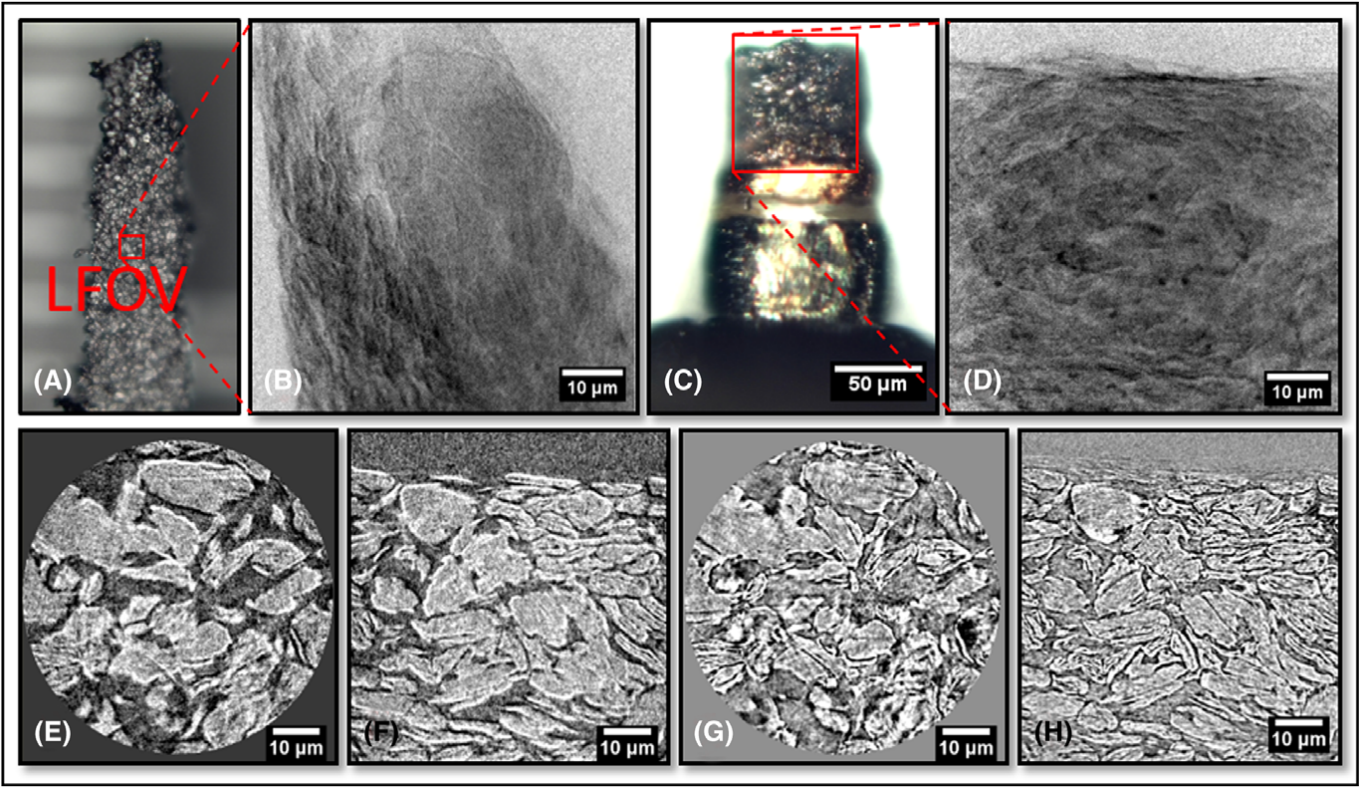
(A) Optical image of traditional mechanical preparation of Li‐ion graphite anode where the red square captures a region that appears to be sufficiently far from the edge of the sample to avoid severe distortion of the microstructure. Note the large amount of material that would be outside the FOV during imaging. (B) Corresponding radiograph of the tip of the sample where the microstructure is distorted from the mechanical preparation technique. (C) Optical image of new laser‐prepared pillar of the graphite anode with copper current collector still attached, (D) Corresponding radiograph. (E), (F) Absorption contrast tomographic slices from the horizontal and vertical planes, respectively. (G), (H) Phase contrast tomographic slices from the horizontal and vertical planes, respectively.

As shown in Figure 9(A), the traditional approach to preparing graphite anode samples comprises crude mechanical sectioning and fixation to the end of a pin, which can cause mechanical damage to the specimen under consideration. The copper current collector is highly attenuating and causes severe problems in terms of reducing signal‐to‐noise ratio and introducing unwanted imaging artefacts.

**Figure 10:**
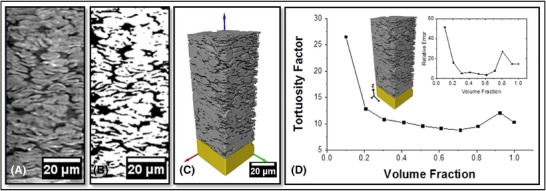
(A) Raw orthoslice from the vertical plane showing the entire depth of the graphite anode where the current collector has been cropped out. (B) Corresponding segmented orthoslice. (C) 3D volume rendering of graphite electrode (grey) on Cu current collector (yellow). (D) RVE Analysis of tortuosity calculated in the through‐plane direction (direction of Li‐ion percolation), with error inset.

Here, the full electrode thickness is captured without being obscured by the current collector (Fig. 9D), and with well‐defined directionality, providing access to calculations of porosity, tortuosity factor, and potentially particle size distribution (PSD) and particle shape anisotropy, which are all relevant to the transport performance of the electrode. An RVE analysis based on tortuosity factor measurements performed in TauFactor is shown in Figure 10(D).

Unfortunately, acceptable convergence in the calculated tortuosity factor is not reached within the volume inspected. This is thought to be due to the large elongation of the particles in the direction parallel to the current collector preventing the full capture of the tortuous pathways. This demonstrates the need for RVE calculations to be performed on the material of interest; further work focusing on stitching together two side‐by‐side datasets is envisaged.

### Case study 4: Shale

After micromachining a coarse pillar of 2 mm in diameter from the bulk substrate, the sample was imaged at a low accelerating voltage of 70 keV (relative to a ROI scan of a bulk sample), with a nominal resolution of approximately 1.05 μm. High data quality was obtained, which revealed a total of four distinguishable phases. Detailed petrographic observations link the four phases to pyrite (brightest), clay matrix (majority), pores and organic matter (darkest) and a grey‐scale attributable to a number of similar density minerals (quartz, feldspar, calcite and dolomite).

**Figure 11:**
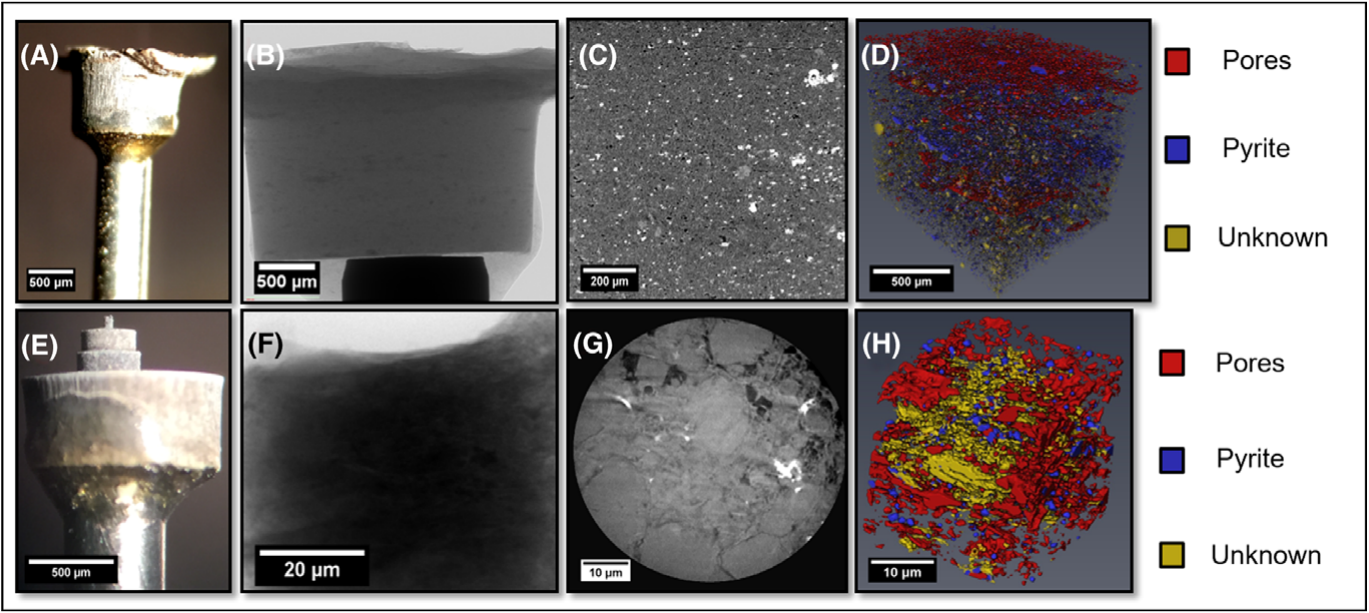
(A) Optical image of coarse pillar for micro‐CT. (B) Corresponding radiograph. (C) Raw orthoslice displaying four phases. (D) 3D reconstruction showing the extra segmentable phase. (E) Optical image of fine pillar for nano‐CT. (F) Corresponding radiograph. (G) Particulate microstructure evident from raw slice. (H) 3D reconstruction of clay particles at high resolution.

A subvolume of 1222 × 1037 × 1061 isotropic voxels (1.05 μm dimensions) was extracted from the full dataset and segmented without filtering, by simple grey‐scale thresholding. An example three‐dimensional reconstruction, with the majority phase transparent, is shown in Figure 11(D), where preferential fracturing in the bedding direction is evident. Because shales are hierarchical structures, a more detailed investigation was carried out at a shorter length‐scale by milling a finer pillar and switching to X‐ray nano‐CT, with a pixel size of 63 nm and 65 μm FOV. The particulate structure becomes visible now (Fig. 11G), providing a deeper insight into the composition of the shale and providing information down to the submicron level on the carbonaceous inclusions, as well as potentially on PSD and particle shape anisotropy. The volume fractions attributable to each phase for both the coarse and fine pillars are shown in Table 6.

**Table 6:** Volume fractions associated with phases identified in shale sample

Phase	Micro‐CT	Nano‐CT
	Volume fraction (%)	
Pore/C	1.6	5.0
Pyrite	2.7	1.8
Cementitious matrix	93.3	85.9
Unknown phase(s)	2.4	7.3

Due to low volume fraction (ca. 1.6%) and a visibly large proportion of isolated pore/carbonaceous (Pore/C) regions, the micro‐CT volume shows no percolation of this phase in any of the three orthogonal directions. However, it should be noted that bedding fractures are likely to be highly heterogeneous and that there will be meso‐ and micro‐pores, which are below the spatial resolution achieved by this X‐ray micro‐CT approach that will remain unidentified in this analysis. Consequently, the true tortuosity/permeability is likely to be inaccessible at this length scale.

A subvolume consisting of 456 × 486 × 501 isotropic (63.1 nm dimension) voxels was extracted from the nano‐CT dataset. A Gaussian filter was applied before simple grey‐scale and watershed thresholding were used to segment the Pore/C phase from all other phases. Simple thresholding, along with erosion and dilation, was used to segment the other three phases. A three‐dimensional reconstruction is shown in Figure 11(H). The bulk porosity was calculated to be 5.0%, which is higher than the 1.6% calculated from the micro‐CT data. It is suggested that this derives from the spatial resolution limit associated with the latter technique; a range of submicrometre Pore/C regions are identifiable when using X‐ray nano‐CT. Moreover, at a higher resolution, a smaller proportion of voxels will encapsulate two or more phases, and hence the so‐called ‘partial volume’ effect may be reduced. The proportion of pyrites is similar, although unlikely to be representative at this scale, given the degree of their heterogeneous distribution. The volume fraction of the ‘unknown’ phase is noticeably higher than in the micro‐CT scan; however, this is explained by the inclusion of one particularly large particle (constituting ∼74% of this phase); without it, the phase volume fraction drops to 1.9% (c.f. 2.4% from micro‐CT). This analysis suggests that a multiscale approach is required when investigating such hierarchical structures and illustrates that the particulate nature of shales is resolvable at this scale.

## Conclusion

The data presented indicate that the laser micro‐machining of samples to give suitable sized pillars of appropriate geometry for X‐ray CT is reliable, versatile and can yield a variety of improvements compared with commonly used sample preparation techniques. It also demonstrates the robustness of the technique, where mechanical integrity and microstructural properties are not significantly affected by its application, exemplifying a number of advantages over competing preparation routes.

In terms of phase segmentation, the laser machining approach enables the segmentation of previously difficult‐to‐distinguish phases, whilst keeping a representative volume, as well as the reduction of accelerating voltages to enhance contrast relative to some bulk samples. With regards to thin films, this micromachining tool can be implemented as an alternative to mechanical approaches (or indeed delamination techniques) so as to give the full thickness required for directional analysis after image processing. Further advantages include the reduction in cost of the fabrication of a range of samples, particularly important for low energy lab‐based X‐ray systems, which often incorporate Zernike phase contrast for low‐Z materials.

Maintaining the microstructure on the nanoscale is particularly beneficial for the investigation of microstructure–property relationships across multiple length‐scales and applies to many different scientific areas of interest. The technique can be applied to low porosity samples, such as shale rocks and other geological materials of interest, through porous composite materials with sinuously connected networks (SOFC electrodes), to particulate‐based structures found in many energy materials of relevance.

The novelty of this approach is derived from the various benefits in the acquired data as a result of tailoring the exact preparation procedure to the characteristics of the investigated materials. This type of laser‐preparation for optimal geometry X‐ray CT samples is thought to be applicable to a wide range of other samples and will hopefully provide access to previously unobtainable information both in the laboratory and at synchrotrons.

## References

[jmi12577-bib-0001] Banhart, J. (2008) Advanced Tomographic Methods in Materials Research and Engineering, Oxford University Press, Oxford, UK.

[jmi12577-bib-0002] Bin, B. , Rukai, Z. , Songtao, W. , Wenjing, Y. , Gelb, J. , Gu, A. , Zhang, X. & Ling, S. (2013) Multi‐scale method of nano(micro)‐CT study on microscopic pore structure of tight sandstone of Yanchang Formation, Ordos Basin. Petr. Explor. Dev. 40, 354–358.

[jmi12577-bib-0003] Cnudde, V. & Boone, M.N. (2013) High‐resolution X‐ray computed tomography in geosciences: a review of the current technology and applications. Earth‐Sci. Rev. 123, 1–17.

[jmi12577-bib-0004] Cooper, S. , Bertei, A. , Shearing, P. , Kilner, J. & Brandon, N. (2016) TauFactor: an open‐source application for calculating tortuosity factors from tomographic data. SoftwareX 5, 203–210.

[jmi12577-bib-0005] Ebner, M. , Chung, D.W. , García, R.E. & Wood, V. (2014) Tortuosity anisotropy in lithium‐ion battery electrodes. Adv. Ener. Mater. 4, 1–6.

[jmi12577-bib-0006] Gelb, J. , Gu, A. , Fong, T. , Hunter, L. , Lau, S. & Yun, W. (2011) A closer look at shale: representative elementary volume analysis with laboratory 3D X‐ray computed microtomography and nanotomography. Proc. SCA 58, 1–8.

[jmi12577-bib-0007] Grew, K.N. , Chu, Y.S. , Yi, J. , Peracchio, A.A. , Izzo, J.R. , Hwu, Y. , De Carlo, F. & Chiu, W.K. (2010a) Non‐destructive nanoscale 3D elemental mapping and analysis of a solid oxide fuel cell anode. J. Electrochem. Soc. 157, B783–B792.

[jmi12577-bib-0008] Grew, K.N. , Peracchio, A.A. , Joshi, A.S. , Izzo, J.R. & Chiu, W.K. (2010b) Characterization and analysis methods for the examination of the heterogeneous solid oxide fuel cell electrode microstructure. Part 1: volumetric measurements of the heterogeneous structure. J. Power Sour. 195, 7930–7942.

[jmi12577-bib-0009] Harris, S.J. & Lu, P. (2013) Effects of inhomogeneities: nanoscale to mesoscale on the durability of li‐ion batteries. J. Phys. Chem. C 117, 6481–6492.

[jmi12577-bib-0010] Hounsfield, G.N. (1973) Computerized transverse axial scanning (tomography): part 1. Description of system. Br. J. Radiol. 46, 1016–1022. 4757352 10.1259/0007-1285-46-552-1016

[jmi12577-bib-0011] Izzo, J.R. , Joshi, A. , Grew, K. , Chiu, W. , Tkachuk, A. , Wang, S. & Yun, W. (2008) Nondestructive reconstruction and analysis of solid oxide fuel cell anodes using X‐ray computed tomography at sub‐50 nm resolution. ECS Transact. 13, 1–11.

[jmi12577-bib-0012] Joos, J. , Ender, M. , Carraro, T. , Weber, A. & Ivers‐Tiffée, E. (2012) Representative volume element size for accurate solid oxide fuel cell cathode reconstructions from focused ion beam tomography data. Electrochimica Acta 82, 268–276.

[jmi12577-bib-0013] Kalender, W.A. (2006) X‐ray computed tomography. Phys. Med. Biol. 51, R29. 16790909 10.1088/0031-9155/51/13/R03

[jmi12577-bib-0014] Kashkooli, A.G. , Farhad, S. , Lee, D.U. , Feng, K. , Litster, S. , Babu, S.K. , Zhu, L. & Chen, Z. (2016) Multiscale modeling of lithium‐ion battery electrodes based on nano‐scale X‐ray computed tomography. J. Power Sour. 307, 496–509.

[jmi12577-bib-0015] Kennouche, D. , Chen‐Wiegart, Y.‐C.K. , Riscoe, C. , Wang, J. & Barnett, S.A. (2016a) Combined electrochemical and X‐ray tomography study of the high temperature evolution of Nickel–Yttria Stabilized Zirconia solid oxide fuel cell anodes. J. Power Sour. 307, 604–612.

[jmi12577-bib-0016] Kennouche, D. , Chen‐Wiegart, Y.‐C.K. , Yakal‐Kremski, K.J. , Wang, J. , Gibbs, J.W. , Voorhees, P.W. & Barnett, S.A. (2016b) Observing the microstructural evolution of Ni‐Yttria‐stabilized zirconia solid oxide fuel cell anodes. Acta Materialia 103, 204–210.

[jmi12577-bib-0017] Laurencin, J. , Quey, R. , Delette, G. , Suhonen, H. , Cloetens, P. & Bleuet, P. (2012) Characterisation of solid oxide fuel cell Ni–8YSZ substrate by synchrotron X‐ray nano‐tomography: from 3D reconstruction to microstructure quantification. J. Power Sour. 198, 182–189.

[jmi12577-bib-0018] Ma, L. , Taylor, K.G. , Lee, P.D. , Dobson, K.J. , Dowey, P.J. & Courtois, L. (2016) Novel 3D centimetre‐to nano‐scale quantification of an organic‐rich mudstone: the Carboniferous Bowland Shale, Northern England. Mar. Petr. Geol. 72, 193–205.

[jmi12577-bib-0019] Maire, E. & Withers, P. (2014) Quantitative X‐ray tomography. Int. Mater. Rev. 59, 1–43.

[jmi12577-bib-0020] Salvo, L. , Cloetens, P. , Maire, E. *et al* (2003) X‐ray micro‐tomography: an attractive characterisation technique in materials science. Nucl. Instr. Meth. Phys. Res. Sect. B: Beam Interact. Mater. Atoms 200, 273–286.

[jmi12577-bib-0021] Sayab, M. , Suuronen, J.‐P. , Molnár, F. , Villanova, J. , Kallonen, A. , O'Brien, H. , Lahtinen, R. & Lehtonen, M. (2016) Three‐dimensional textural and quantitative analyses of orogenic gold at the nanoscale. Geology 44, 739–742.

[jmi12577-bib-0022] Shearing, P. , Bradley, R. , Gelb, J. , Lee, S. , Atkinson, A. , Withers, P. & Brandon, N. (2011) Using synchrotron X‐ray nano‐CT to characterize SOFC electrode microstructures in three‐dimensions at operating temperature. Electrochem. Solid‐State Lett. 14, B117–B120.

[jmi12577-bib-0023] Shearing, P. , Bradley, R. , Gelb, J. , Tariq, F. , Withers, P. & Brandon, N. (2012) Exploring microstructural changes associated with oxidation in Ni–YSZ SOFC electrodes using high resolution X‐ray computed tomography. Solid State Ionics 216, 69–72.

[jmi12577-bib-0024] Shearing, P. , Brett, D. & Brandon, N. (2013) Towards intelligent engineering of SOFC electrodes: a review of advanced microstructural characterisation techniques. Int. Mater. Rev. 55, 347–363.

[jmi12577-bib-0025] Shearing, P. , Gelb, J. , Yi, J. , Lee, W.‐K. , Drakopolous, M. & Brandon, N. (2010) Analysis of triple phase contact in Ni–YSZ microstructures using non‐destructive X‐ray tomography with synchrotron radiation. Electrochem. Commun. 12, 1021–1024.

[jmi12577-bib-0026] Shearing, P.R. , Gelb, J. & Brandon, N. (2009) Characterization of SOFC electrode microstructure using nano‐scale X‐ray computed tomography and focused ion beam techniques: a comparative study. ECS Transact. 19, 51–57.

[jmi12577-bib-0027] Taiwo, O.O. , Finegan, D.P. , Eastwood, D.S. *et al* (2016a) Comparison of three‐dimensional analysis and stereological techniques for quantifying lithium‐ion battery electrode microstructures. J. Microsc. 263 **(** 3 **)**, 1–13. 10.1111/jmi.12389 PMC4999027 26999804

[jmi12577-bib-0028] Taiwo, O.O. , Finegan, D.P. , Gelb, J. , Holzner, C. , Brett, D.J. & Shearing, P.R. (2016b) The use of contrast enhancement techniques in X‐ray imaging of lithium–ion battery electrodes. Chem. Eng. Sci. 154, 27–33.

[jmi12577-bib-0029] Tkachuk, A. , Duewer, F. , Cui, H. , Feser, M. , Wang, S. & Yun, W. (2007) X‐ray computed tomography in Zernike phase contrast mode at 8 keV with 50‐nm resolution using Cu rotating anode X‐ray source. Zeitschrift für Kristallographie 222, 650–655.

